# Nature Studies in Macedonia

**Published:** 1916-12-16

**Authors:** 


### NATURE STUDIES IN MACEDONIA.

BY AN OFFICER OF THE R.A.M.C. AND THE AUTHOR OF "NATURE NOTES."

On the hills that bound the Struma Valley, at a height of fifteen or sixteen hundred feet above the sea, a most interesting spider has her home. I say "her home," for that I shall attempt to describe makes it almost certain that it is the female I have been watching. I have no idea what the male is like. I do not see any sign of his presence. Since it is well known that in certain varieties of spiders the amorous male approaches his love only at the risk of his life, maybe in this variety the loving husband gets no chance to stray from his wife's side (inside). The nest is a hole in the ground about as large as will admit the first finger and about five inches deep. The sides of this tunnel are smooth, and the mouth of it is continued above the level of the earth by the binding together with spider's thread of bits of growing grass or bents and earth. The tube and tunnel thus made is about five inches in length, and straight from the mouth to a curve which continues the tunnel on into a chamber. Light penetrates to the curve, but the spider is hidden from view when in the recess. She is a large spider, with bright eyes and an uncannily intelligent-looking face. Her body is grey and as large as the top joint of the little finger. The top of the abdominal segment is a bright orange colour. Her legs are unusually long.

I was first attracted to her home by observing a sudden movement in the grass and the disappearance of a dirty white object that I had not observed till it was in motion. On investigation I discovered the hole and waited, looking down the tunnel. After a minute or two I saw the spider's legs come into view out of the recess at the end of the tunnel. A pause, and then she cautiously climbed half-way up the tunnel. Another pause, and she slowly reached the entrance and sat crouched in it, perfectly still, her black eyes glistening brightly, as if she were scouting for danger. A little while and she turned half-left, so that her right side was towards me, and then, after another pause, she turned half-left again, and brought into view something of a yellowish parchment white colour. Another turn and she was head down in the tunnel, and the white object was displayed as a round ball of spider web, similar to the cocoons made by many moths, presumably full of eggs or perhaps young spiders. This object was protruded right out of the entrance of the tunnel and held in the sunshine by two legs and the abdominal segment of the spider. A slight movement on my part caused the spider to slip quickly down the tunnel, taking herself and her young out of sight into the recess. She reappeared and went through the same performance in a minute or two. Further search revealed two more such nests in the same acre of ground. Knowing their positions I can now go quietly and watch the balls of young or eggs as they are held by the devoted parent in the rays of the sun. The spiders take advantage of all bright sunshine to expose their eggs. In the cold of early morning and the evening they keep them in the recess of the tunnel.

During the battle of September 15 I was surprised to see two hawks of a large size and chestnut colour, with yellow beaks and a white band of feathers at the root of the tail, serenely hawking about over the crops not 400 yards behind a line on which we had several batteries in action. They took no heed at all of the report of our guns, nor of the sounds of the bursts of the shells sent in reply by the enemy. I watched them work over the same area for over an hour. I cannot name the species. Later I moved my position and passed by a heavy gun hidden in the plain. Close by it I saw a bird fluttering curiously on the ground. I went to pick it up and saw it was a crested lark, common here, and similar to, if not identical with, the English lark. It had no wing nor leg broken, yet it could neither run nor fly; but fluttered and pushed with its legs along the ground rapidly on an erratic course, much as a partridge shot through the brain will flutter. Yet it appeared to be conscious, for it seemed to make progressive efforts to escape me. I caught it and examined it. I could see no injury. One of the gun team told me he thought it had been knocked over by the blast of the explosion of the gun. I think he was right.

Since I wrote the above, on September 18, the devotion of two of my spiders has been rewarded with a large family of minute spiders alive. The mothers now sit at the entrance of their holes with dozens of little tiny spiders on their backs. The little ones cluster so thickly that they give the mother a hairy appearance. Occasionally one adventurous babe will run across from one side of its mother to the other. They are growing rapidly: it is September 21 to-day, and in the two days of their existence I see a marked increase in size. Yet I cannot see that they feed on anything. Nor have I seen the mother catch any form of insect, either since the hatching of the young or before that event.

